# Rhamnolipids Regulate Lipid Metabolism, Immune Response, and Gut Microbiota in Rats

**DOI:** 10.3389/fnut.2022.886256

**Published:** 2022-04-28

**Authors:** Bing Zhang, Songke Qin, Yanping Wu, Ruiqiang Zhang, Yinglei Xu, Caimei Yang

**Affiliations:** Key Laboratory of Applied Technology on Green-Eco-Healthy Animal Husbandry of Zhejiang Province, Zhejiang Provincial Engineering Laboratory for Animal Health and Internet Technology, College of Animal Science and Technology, College of Veterinary Medicine, Zhejiang Agriculture and Forestry University, Hangzhou, China

**Keywords:** rhamnolipids, lipid metabolism, immune response, gut microbiota, Sprague-Dawley rats

## Abstract

**Objectives:**

Gut microbes influence lipid metabolism and immune responses that are key features of metabolic disorders. This study examined effects of bacterial rhamnolipids (RLS) on lipid metabolism, immune response, and gut microbiota in rats.

**Methods:**

Twenty-four Sprague-Dawley rats were randomly divided into three groups and gavage-fed for seven weeks with normal saline (NCO group), 50 mg/kg bw RLS (RLS1 group), and 100 mg/kg bw RLS (RLS2 group).

**Results:**

Compared with those of the NCO group, the RLS1 and RLS2 groups showed significantly decreased fat weight, relative fat weight, and adipocyte size (*P* < 0.05). Furthermore, RLS1 and RLS2 significantly decreased concentrations of triglycerides, low-density lipoprotein-cholesterol, and non-esterified fatty acids and increased high-density lipoprotein-cholesterol levels (*P* < 0.05). However, the total cholesterol content among the three groups (*P* > 0.05) were not significantly different. Serum concentrations of interleukin-1β, interleukin-6, and tumor necrosis factor-α were significantly lower in the RLS2 group than those in the NCO group (*P* < 0.05). The relative mRNA expression of fatty acid synthase was significantly decreased, while those of carnitine palmitoyltransferase-1, carnitine palmitoyltransferase-2, and peroxisome proliferator-activated receptor-gamma coactivator-1α were significantly increased in the RLS2 group compared with those in the NCO group (*P* < 0.05). Moreover, the relative abundances of *Lactobacillus, Roseburia, Ruminococcus-1*, and *Parabacteroides* were significantly higher in the RLS2 group than those in the NCO group (*P* < 0.05).

**Conclusion:**

Our findings suggest that RLS reduces fat deposition, inhibits inflammation, regulates intestinal flora, and promotes the proliferation of beneficial bacteria in rats.

## Introduction

In mammals, lipid metabolism is a key player in several metabolic disorders, including diabetes, metabolic syndromes, and systemic diseases such as cancer (1). Therefore, lipid metabolism disorders are among the most important types of metabolic abnormalities and cannot be ignored. Lipid metabolism is associated with immune responses and inflammatory processes in mammals (2). However, the exact mechanism underlying the interaction between abnormal lipid metabolism and inflammatory responses remains unclear. Furthermore, obvious correlations between the proportions of specific gut bacteria taxa and lipid levels indicate a role of intestinal microorganisms in regulating host lipid metabolism (3). The gut microbiome can mechanistically affect host lipid levels (4–6). Another possible underlying mechanism whereby intestinal microorganisms affect lipid metabolism may involve fermentation of undigested carbohydrates (7). Moreover, intestinal microbiota may possibly produce intermediate precursors that are further metabolized to products that directly affect lipid levels of the host (8).

Rhamnolipids (RLS) are glycolipids produced by *Pseudomonas aeruginosa* and are composed of one or two rhamnose molecules linked to one or two fatty acid alkyl chains (9). Several useful properties of RLS biosurfactants such as surface activity, emulsifying properties, biodegradability, and low toxicity have been exploited in the processing industry (10). Furthermore, RLS have broad-spectrum antimicrobial activity against both gram-positive and gram-negative bacteria (11–13). RLS also participate in immune defense responses in plants, animals, and humans and play a significant role in the treatment of various immune diseases (14). In our previous study, RLS improved growth performance, regulated immune responses, increased the abundance of intestinal flora, and promoted the proliferation of beneficial bacteria in broiler chickens (15). Gut microbes provide essential nutrients, affect lipid metabolism and levels in blood and tissues, and modulate the host immune system (16). Therefore, in this study, we evaluated effects of RLS on lipid metabolism and inflammatory factors in rats to provide a theoretical basis for the subsequent in-depth study on the mechanism by which RLS regulates lipid metabolism and inflammatory responses.

## Materials and Methods

### Animals and Study Design

All experiments were conducted in accordance with the Guidelines for the Care and Use of Laboratory Animals of Zhejiang Agriculture and Forestry University and were approved by the Animal Ethics Committee of Zhejiang Agriculture and Forestry University (SYXKzhe 2019-054).

RLS (purity 99.9%, mixture) used in the present work were provided by Zhejiang Vegamax Biological Technology Co. Ltd. (Huzhou, China). RLS were weighed according to the body weight of the rat, put into a 1.5 mL centrifuge tube, and 1 mL phosphate buffered saline (PBS) was added to prepare RLS suspension.

A total of 24 adult male Sprague-Dawley rats weighing 200 ± 20 g were purchased from Zhejiang Academy of Medical Sciences (Hangzhou, China). Prior to the experiment, all rats were housed in an independently ventilated cage with free access to food and water for 1 week to acclimatize to the environment. Rats were randomly divided into three treatment groups (*n* = 8 per group). The control group (NCO group) was gavage-fed normal saline. The RLS1 and RLS2 groups were gavage-fed 50 and 100 mg/kg bw RLS, respectively. Gavage feeding was performed for seven weeks.

### Sample Collection

At the end of the experiment, each rat was weighed and euthanized. Blood was obtained from the abdominal aorta after rats were anesthetized with sodium pentobarbital. Serum samples were collected by centrifugation at 6,000 rpm for 10 min and stored at−80°C. Inguinal, epididymal, and subscapular adipose tissues were weighed and preserved in 4% formalin or were immediately frozen in liquid nitrogen. Liver and colonic contents were collected and frozen at -80°C.

### Body Weight and Fat Weight

Body weight and inguinal, epididymal, and subscapular fat weights of each rat were measured by electronic analytical balance (BSA124S, Sartorius, Beijing, China) at the end of the experiment. Relative fat weight was calculated as: relative weight = weight of specific adipose tissue/weight of the rat.

### Serum Biochemical Indicators

Serum biochemical parameters, such as levels of total cholesterol (TC), triglyceride (TG), low-density lipoprotein-cholesterol (LDL-C), high-density lipoprotein-cholesterol (HDL-C), non-esterified fatty acid (NEFA), interleukin-1β (IL-1β), interleukin-6 (IL-6), interleukin-10 (IL-10), and tumor necrosis factor-α (TNF-α), were measured according to the manufacturer's instructions (Nanjing Jiancheng Bioengineering Institute, Nanjing, China).

### Adipose Tissue Morphology

Inguinal, epididymal, and subscapular adipose tissues were fixed with 4% formaldehyde for at least 24 h. Samples were dehydrated using graded ethanol solutions, cleared using xylol, and embedded in paraffin. Sections (5 μm thick) were washed with xylene to remove residual paraffin, rinsed with different concentrations of alcohol, and finally stained with hematoxylin-eosin (HE). Tissue sections from each group were examined under an optical microscope (Eclipse Ci, Nikon, Japan). Three visual fields were randomly selected to capture photographs, and the magnification of the view was 200×. The average adipocyte size was measured using the ImageJ software (National Institute of Mental Health, Bethesda, USA).

### Real-Time Quantitative PCR

Total RNA in the liver was extracted using the RNAiso reagent (Takara Bio, Inc., Japan). A Nano-300 micro-spectrophotometer (AllSheng Instruments Co. Ltd., Hangzhou, China) was used to measure the quality and concentration of the isolated RNA. RNA samples were reverse transcribed using the PrimeScript RT Master Mix reagent kit (RR047A, Takara Bio, Inc., Japan) with a gDNA eraser. β-actin was used as an internal control. Primer sequences used in this study were synthesized by TSINGKE Biological Technology (Hangzhou, China), and are listed in Table 1. The CFX96 Real-Time System (Bio-Rad, Singapore) was used to perform PCR using an RT-PCR kit (RR420A, Takara Bio, Inc., Japan). The reaction was performed as follow: denaturation at 95°C for 30s, followed by 40 cycles (95°C for 5 s and 60°C for 30 s). The crossing threshold (Ct) values of different treatments were obtained to calculate relative mRNA levels using the 2^−ΔΔCt^ method. The mRNA level of each target gene in the control group was considered to represent the baseline level, with an assigned fold change of one.

**Table 1:** Sequences of the oligonucleotide primers used for quantitative real-time PCR.

**Gene^a^**	**GenBank accession number**	**Sequence (5^**′**^-3^**′**^)**
*β-actin*	NM_031144	Forward: GATTACTGCCCTGGCTCCTA
		Reverse: TCATCGTACTCCTGCTTGCT
*ACC*	NM_022193.1	Forward: TGAAGGGCTACCTCTAATG
		Reverse: TCACAACCCAAGAACCAC
*FAS*	NM_017332	Forward: AGCCGCCGACCAGTAT
		Reverse: CACAGACACCTTCCCATCA
*Srebf-1c*	AF286470	Forward: GGAGCCATGGATTGCACATT
		Reverse: AGGAAGGCTTCCAGAGAGGA
*CPT-1*	NM_009948	Forward: CAACACTACACGCATCCC
		Reverse: GAAAGATTTGTCAAACCACC
*CPT-2*	NM_012930.1	Forward: TGACCAGTGAGAACCGAGAT
		Reverse: GGCAGAGGCAGAAGACAG
*PGC-1α*	NC_005113.4	Forward: TGGAGTCCACGCATGTGAAG
		Reverse: CGCCAGCTTTAGCCGAATAG

a *ACC, acetyl-CoA carboxylase; FAS, fatty acid synthase; Srebp-1c, sterol regulatory-element binding proteins-1c; CPT-1, carnitine palmitoyltransferase-1; CPT-2, carnitine palmitoyltransferase-2; PGC-1α, peroxisome proliferator-activated receptor-gamma coactivator-1α*.

### Colonic Microflora

Total genomic DNA was extracted from colonic samples of rats using a DNA extraction kit, and the DNA concentration was determined using NanoDrop 2000 and agarose gel electrophoresis. Genomic DNA was used as a template for PCR amplification, which was performed using barcoded primers and Tks Gflex DNA Polymerase (Takara Bio). The V3–V4 region of the 16s rRNA gene was analyzed using specific primers with the following sequences: 343F (5′-TACGGRAGGCAGCAG-3′) and 798R (5′-AGGGTATCTAATCCT-3′). The amplification quality was examined using gel electrophoresis. PCR products were purified using AMPure XP beads (Agencourt) and amplified in another round of PCR. After purification, the final amplification was quantified using a Qubit dsDNA detection kit. Equal amounts of purified amplification products were pooled for subsequent sequencing. Library construction and sequencing were performed by the Shanghai OE Biotech. Co., Ltd (Shanghai, China).

### Statistical Analysis

One-way analysis of variance was performed using SPSS (version 25.0; SPSS Inc., USA) and GraphPad Prism 8.0 (GraphPad Software Inc., USA). Values are expressed as mean. Differences among treatments were examined using the least significant difference test. Significance levels: ^*^ *P* < 0.05, ^**^ *P* < 0.01, ^***^ *P* < 0.001.

## Results

### Relative Fat Weight

The relative weight of epididymal fat was significantly lower in RLS1 rats than that in NCO rats (*P* < 0.05). Moreover, the relative weights of the inguinal, epididymal, and subscapular adipose tissues were significantly lower in the RLS2 group than that in the control group (*P* < 0.05) (Table 2).

**Table 2:** Effects of RLS on relative fat weight in rats^1^.

**Items**	**Treatments^2^**			**SEM^3^**	***P-*value**
	**NCO**	**RLS1**	**RLS2**		
Inguinal relative fat weight (%)	2.91^a^	2.64^a^	2.08^b^	0.12	0.010
Epididymal relative fat weight (%)	2.37^a^	1.62^b^	1.35^b^	0.13	<0.001
Subscapular relative fat weight (%)	0.20^a^	0.17^a^	0.15^b^	0.01	0.002

1 *Data are expressed as mean, n = 8. Values in the same line with different superscripts are significantly different (P < 0.05), while those with the same superscripts are not significantly different (P > 0.05)*.

2 *NCO, rats were gavage-fed normal saline; RLS1, rats were gavage-fed 50 mg/kg·bw RLS; RLS2, rats were gavage-fed 100 mg/kg·bw RLS*.

3 *Pooled SEM values*.

### Adipocyte Size

**Figure 1:**
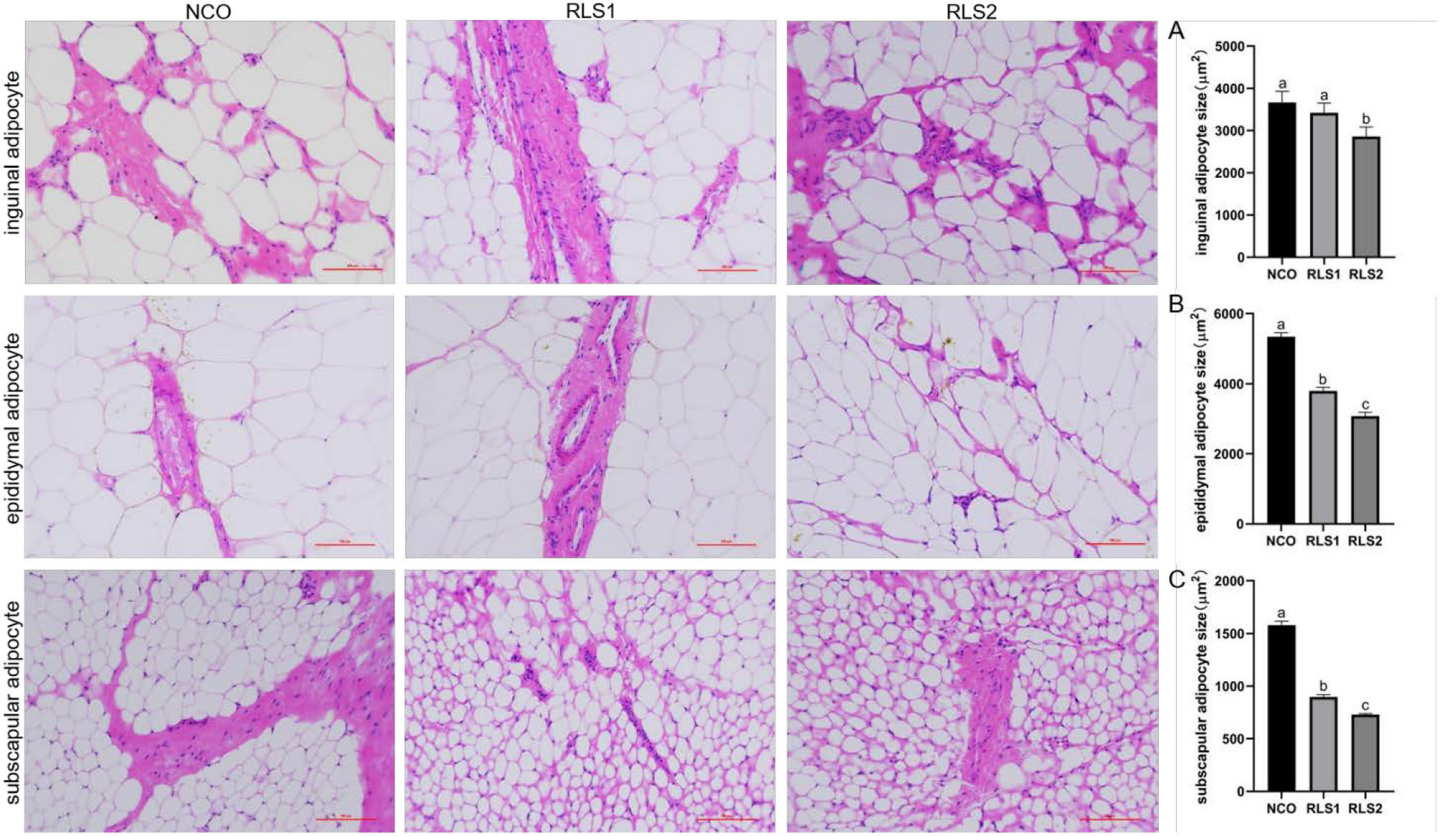
Effects of RLS on adipocyte size in rats. NCO, rats were gavage-fed normal saline; RLS1, rats were gavage-fed 50 mg/kg·bw RLS; RLS2, rats were gavage-fed 100 mg/kg·bw RLS. HE staining (200×). The histograms represent quantification of the adipocyte sizes. Values are expressed as the mean ± SEM, *n* = 8. Different letters represent a significant difference of *P* < 0.05. Part labels represent standard error for the sample mean (SEM).

HE staining demonstrated that the size of inguinal adipocytes in the RLS2 group was significantly smaller than that in the NCO group (*P* < 0.05). However, the dosage of RLS administered in the RLS1 group had no significant impact on inguinal adipocyte size (*P* > 0.05). A significant reduction in epididymal and subscapular adipocyte sizes was observed in both RLS1 and RLS2 rats (*P* < 0.05). Furthermore, adipocyte size was significantly smaller in the RLS2 group than in the RLS1 (*P* < 0.05) (Figure 1).

### Serum Lipid Levels

**Figure 2:**
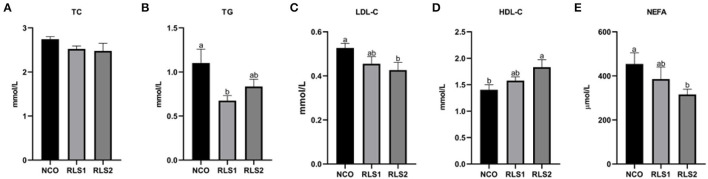
Effects of RLS on serum lipid levels in rats. NCO, rats were gavage-fed normal saline; RLS1, rats were gavage-fed 50 mg/kg·bw RLS; RLS2, rats were gavage-fed 100 mg/kg·bw RLS. TC, total cholesterol; TG, triglyceride; LDL-C, low-density lipoprotein-cholesterol; HDL-C, high-density lipoprotein-cholesterol; NEFA, non-esterified fatty acid. Values are expressed as the mean ± SEM, *n* = 8. Different letters represent a significant difference of *P* < 0.05. Part labels represent standard error for the sample mean (SEM).

The TG level in the RLS1 group was significantly lower than that in the control group (*P* < 0.05). The RLS2 group induced a significant decrease in LDL-C and NEFA levels, and a significant increase in HDL-C levels (*P* < 0.05). However, TC levels did not differ significantly among the three groups (*P* > 0.05) (Figure 2).

### Lipid Synthesis and Degradation-Related Gene Expression

**Figure 3:**
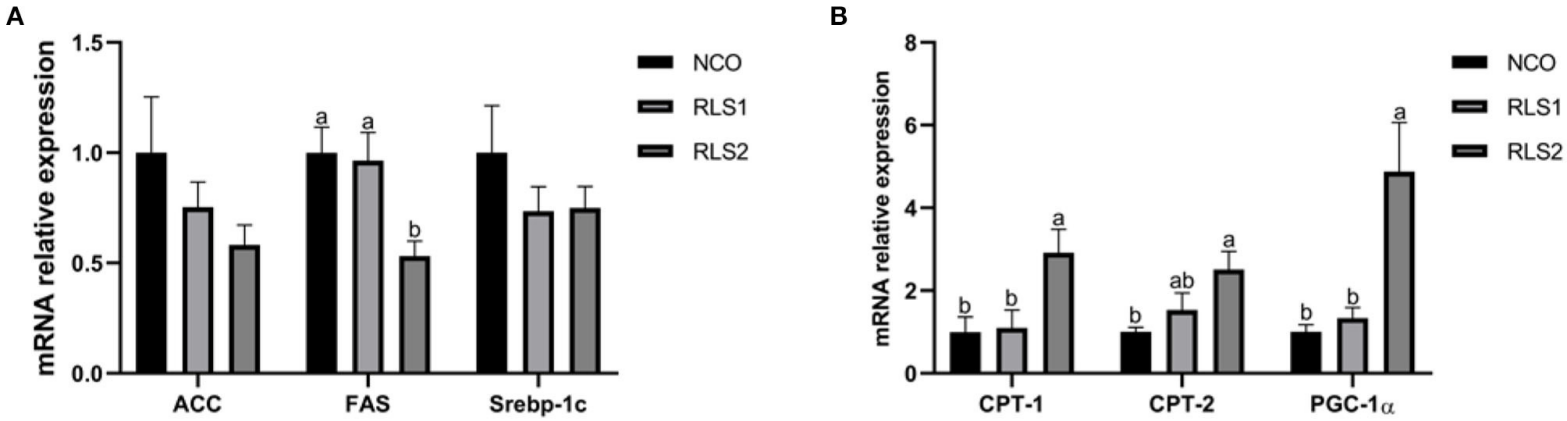
Effects of RLS on the expression of lipid synthesis and degradation-related genes in the liver of rats. NCO, rats were gavage-fed normal saline; RLS1, rats were gavage-fed 10 mg RLS / 200 g body weight; RLS2, rats were gavage-fed 20 mg RLS / 200 g body weight. ACC, acetyl-CoA carboxylase; FAS, fatty acid synthase; Srebp-1c, sterol regulatory-element binding proteins-1c; CPT-1, carnitine palmitoyltransferase-1; CPT-2, carnitine palmitoyltransferase-2; PGC-1α, peroxisome proliferator-activated receptor-gamma coactivator-1α. Values are expressed as the mean ± SEM, *n* = 8. Different letters represent a significant difference of *P* < 0.05. Part labels represent standard error for the sample mean (SEM).

Compared with those of the NCO group, relative mRNA levels of the lipid synthesis-related gene fatty acid synthase (*FAS*) were significantly decreased in the RLS2 group (*P* < 0.05). We also observed a decreasing trend in the expression of the lipid synthesis-related genes— acetyl-CoA carboxylase (*ACC*) and sterol regulatory-element binding proteins-1c (*Srebp-1c*) (*P* > 0.05). Additionally, expressions of lipid degradation-related genes, namely carnitine palmitoyltransferase-1 and 2 (*CPT-1 and CPT-2*), and peroxisome proliferator-activated receptor-gamma coactivator-1α (*PGC-1*α), were significantly higher in RLS2 rats than those in NCO rats (*P* < 0.05). However, differences in lipid degradation-related gene expression were not significant between the RLS1 and NCO groups (*P* > 0.05) (Figure 3).

### Serum Inflammatory Cytokines

**Figure 4:**
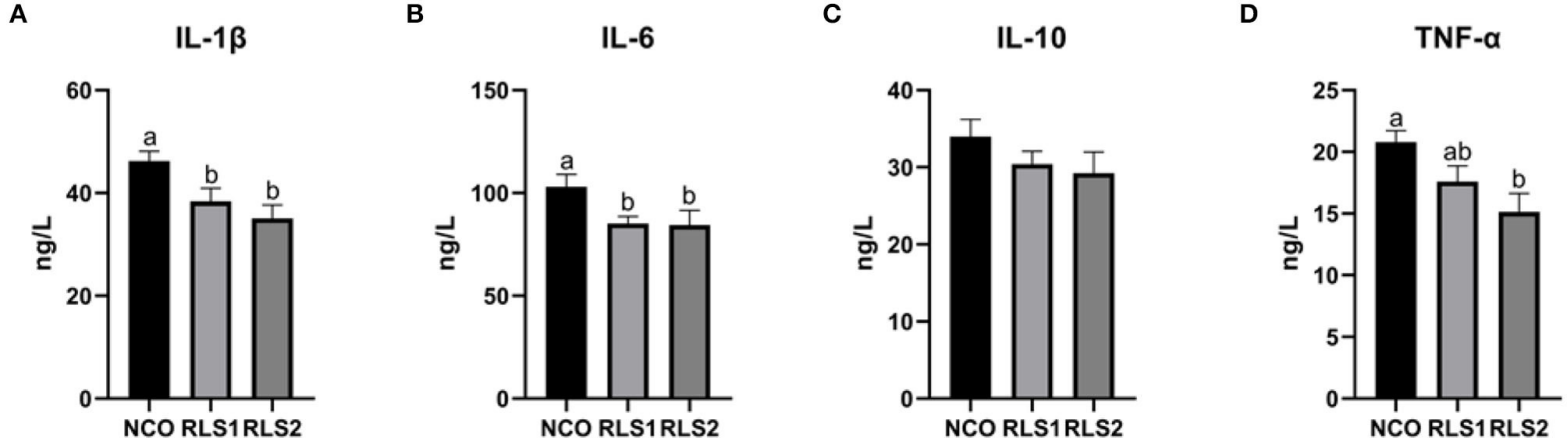
Effects of RLS on serum inflammatory cytokine levels in rats. NCO, rats were gavage-fed normal saline; RLS1, rats were gavage-fed 10 mg RLS / 200 g body weight; RLS2, rats were gavage-fed 20 mg RLS / 200 g body weight. IL-1β, interleukin-1β; IL-6, interleukin-6; IL-10, interleukin-10; TNF-α, tumor necrosis factor-α. Values are expressed as the mean ± SEM, *n* = 8. Different letters represent a significant difference of *P* < 0.05. Part labels represent standard error for the sample mean (SEM).

Serum IL-1β and IL-6 concentrations significantly decreased in the RLS1 and RLS2 groups (*P* < 0.05). Moreover, RLS2 significantly reduced the TNF-α concentration in the serum of rats compared with NCO group (*P* < 0.05). However, no significant alterations in the serum levels of anti-inflammatory cytokines (IL-10) were observed (*P* > 0.05) between the three groups (Figure 4).

### Colonic Microflora

**Figure 5:**
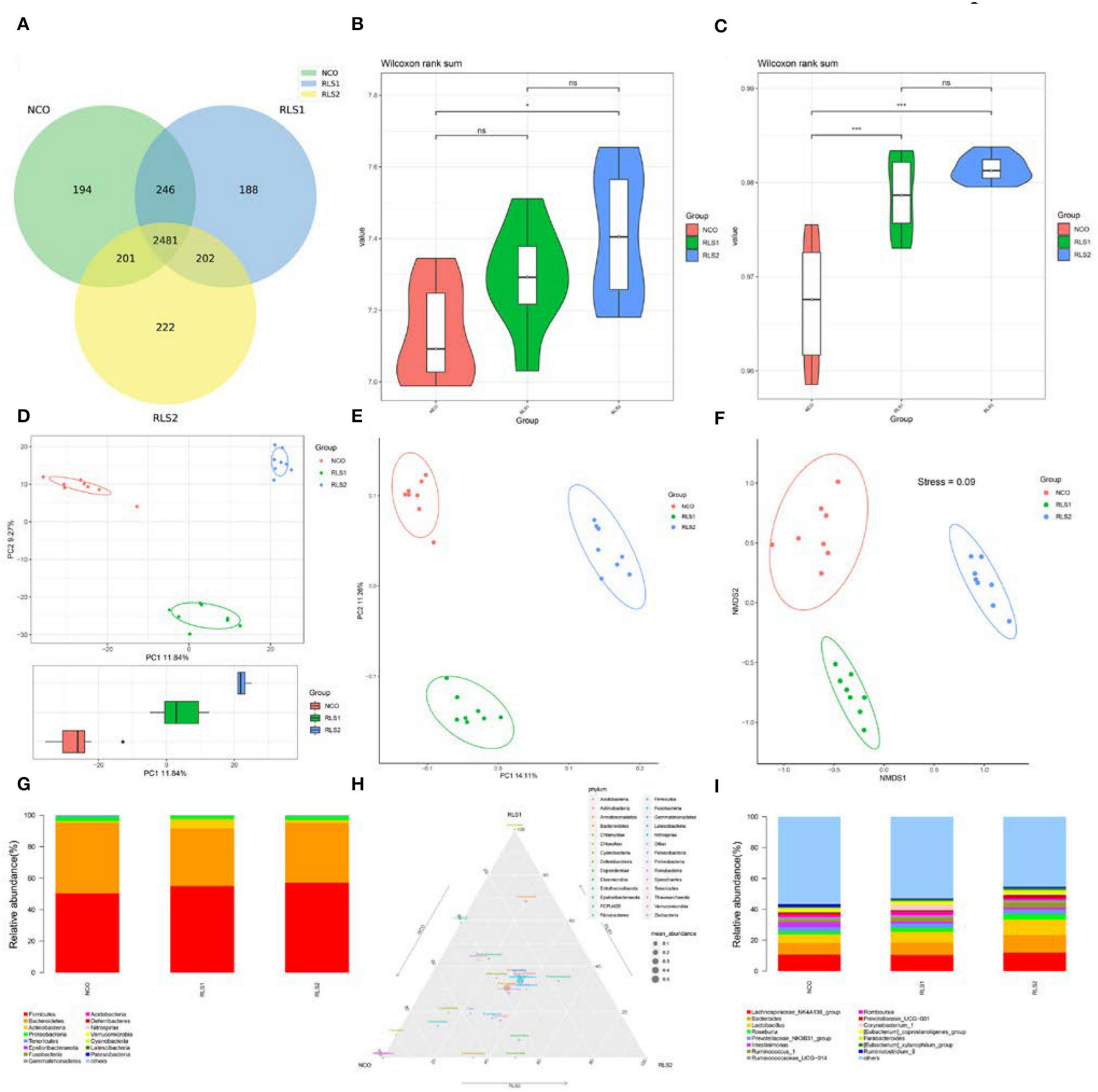
Effects of RLS on the colonic microflora in rats. NCO, rats were gavage-fed normal saline; RLS1, rats were gavage-fed 10 mg RLS / 200 g body weight; RLS2, rats were gavage-fed 20 mg RLS / 200 g body weight. **(A)** Venn diagram. **(B)** Shannon index. **(C)** Simpson index. **(D)** Principal component analysis (PCA). **(E)** Principal coordinate analysis (PCoA). **(F)** Non-metric multidimensional scaling (NMDS). **(G)** The microbiota composition at phylum level. **(H)** Ternary phase diagram of the dominant phyla. **(I)** The microbiota composition at genus level. Data are shown as mean ± SEM and analyzed by one-way ANOVA LSD test (*n* = 8 in each group). * represents *P* < 0.05, *** represents *P* < 0.001, ns represents not-significant.

There were 2,481 common operational taxonomic units (OTUs) shared across the three groups and 194, 188, and 222 unique OTUs were present in the NCO, RLS1, and RLS2 groups, respectively (Figure 5A). Compared with those of the NCO group, the Shannon and Simpson (α-diversity) indices were significantly increased in the RLS2 group (*P* < 0.001 each) (Figures 5B,C). The Simpson index in the RLS1 group was significantly higher (*P* < 0.001) than that in the NCO group (Figure 5C). Colonic microflora among the three groups differed, as indicated by the principal component analysis and principal coordinate analysis (Figures 5D,E). In addition, non-metric multidimensional scaling plots indicated a greater distance between the NCO, RLS1, and RLS2 groups (Figure 5F). Firmicutes, Bacteroidetes, Actinobacteria, Proteobacteria, and Tenericutes were the dominant bacterial phyla in all the treatment groups (Figures 5G,H). Meanwhile, *Lachnospiraceae, Bacteroides, Lactobacillus, Romboutsia*, and *Prevotellaceae* were highly abundant in all the RLS groups, as shown in Figure 5I.

**Figure 6:**
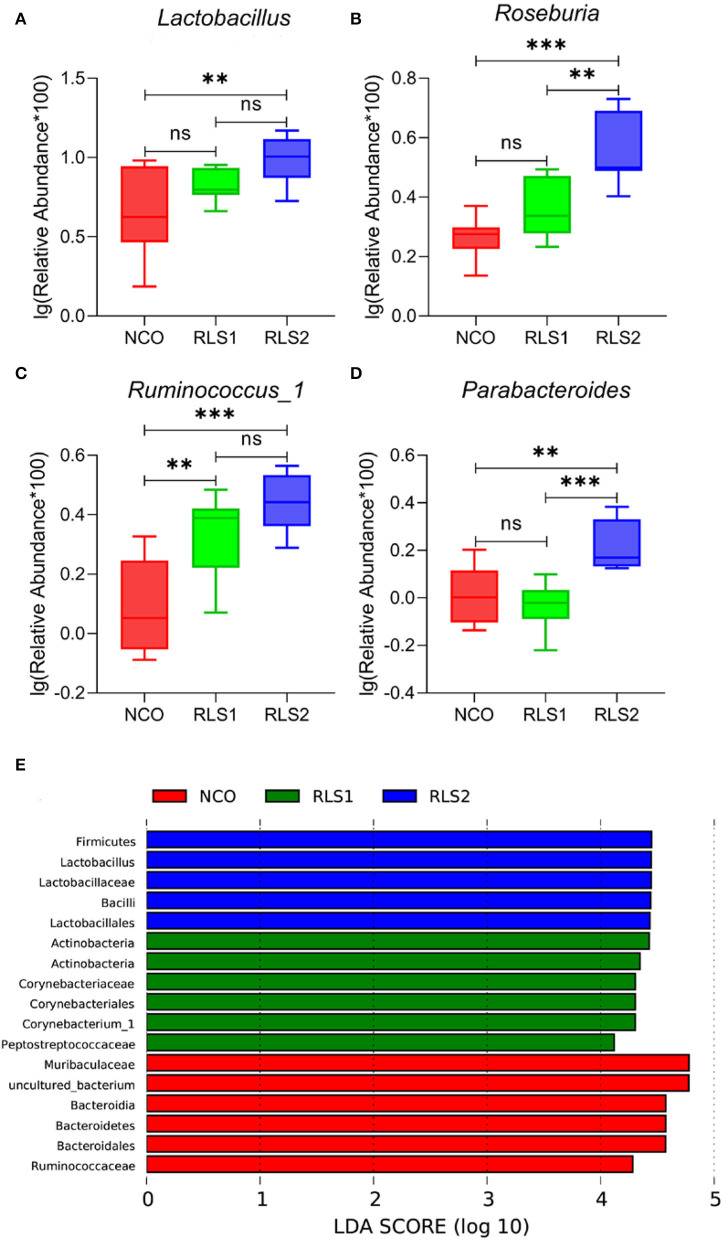
Significant genera and LEfSe analysis. NCO, rats were gavage-fed normal saline; RLS1, rats were gavage-fed 10 mg RLS / 200 g body weight; RLS2, rats were gavage-fed 20 mg RLS / 200 g body weight. **(A–D)** The significant genera in the three treatment groups. **(E)** Histogram of LDA scores for taxonomic biomarkers by LEfSe analysis. LDA scores (log 10) > 2 indicate enriched taxa in cases. Data are shown as mean ± SEM and are analyzed by one-way ANOVA LSD test (*n* = 8 in each group). ** represents *P* < 0.01, *** represents *P* < 0.001, ns represents not-significant.

Differences at the genus level are shown in panels A–D of Figure 6. Rats in the RLS1 group had a higher abundance of *Ruminococcus-1* than those in the NCO group (*P* < 0.01). Meanwhile, rats in the RLS2 group had a higher abundance of *Lactobacillus, Roseburia, Ruminococcus-1*, and *Parabacteroides spp*. than those in rats of the NCO group (*P* < 0.01, *P* < 0.001, *P* < 0.001, and *P* < 0.01, respectively). At the genus level, LEfSe analysis showed that *Firmicutes* and *Lactobacillus* were abundant in the RLS2 group, *Actinobacteria* and *Corynebacteriaceae* in the RLS1 group, and *Muribaculaceae* and uncultured bacteria in the NCO group (Figure 6E).

## Discussion

Impaired adipose tissue function is a major contributor to lipid metabolism disorders (17, 18). Adipose tissue is the main site of fat storage in the body and is involved in the de novo synthesis of fat and oxidative decomposition of fatty acids, which is essential to maintain fat metabolism balance (19). Adipose tissue stores energy mainly in the form of triglycerides (TG). When fat intake exceeds the storage capacity of adipocytes, lipids cannot be effectively utilized by the body, leading to increased lipid levels (20). Furthermore, excessive deposition of lipids in adipose tissues causes adipocytes to secrete large amounts of pro-inflammatory cytokines and recruit a large number of white blood cells into the adipose tissue (21, 22). As the number of infiltrating leukocytes increases, pro-inflammatory cytokines produced by adipose tissues continue to be released into the circulatory system, leading to mild systemic chronic inflammation (21, 22). This inflammation further aggravates any existing metabolic disorders in the body and promotes the development of common metabolic diseases such as cardiovascular disease and type II diabetes (21, 22). Therefore, screening and using active substances to regulate fat metabolism and inflammation are extremely important for the treatment and prevention of diseases related to metabolic disorders and for the protection of animal and human health. In this study, RLS regulated lipid metabolism and immune responses by inhibiting the expression of lipid synthesis-related genes, promoting the expression of lipid decomposition-related genes, and decreasing the secretion of inflammatory factors in rats. Therefore, this study provided a theoretical basis for the subsequent in-depth study on the mechanism underlying the regulation of lipid metabolism and inflammatory responses by RLS.

Fat weight and adipocyte size are important parameters for evaluating lipid accumulation in adipose tissues (23). Lipid metabolism disorders lead to excessive accumulation of lipids in adipocytes, resulting in increased adipocyte size (24). Our results indicated that RLS decreased the weight of inguinal, epididymal, and subscapular adipose tissues. Additionally, adipocyte sizes in different adipose tissues in RLS-fed rats was significantly decreased compared with those in rats of the NCO group. These results suggest that RLS treatment can significantly inhibit lipid accumulation in different adipose tissues in rats. The main reason for this may be that RLS modulates fat deposition by modulating the expression of lipid metabolism-related genes. However, the effects of RLS on fat metabolism are scarcely reported in literature.

Dyslipidemia is a disorder of lipid metabolism and is defined by higher levels of TC, TG, and LDL-C as well as lower concentrations of HDL-C in the serum (25). Suarez-Sanchez et al. (26) reported that a decrease in lipid levels of serum is associated with a reduced risk of metabolic diseases. Furthermore, Frohnert et al. (27) showed that elevated serum NEFA levels can increase the risk of adiposity, insulin resistance, and angina pectoris. In our study, RLS reduced levels of TC, TG, and LDL-C, and increased the HDL-C and NEFA levels, indicating that RLS improve the serum lipid profiles of rats.

To further clarify the mechanism underlying the inhibitory effects of RLS on lipid accumulation in rats, we examined gene expression levels of key factors regulating fat metabolism in the livers of rats. The maintenance of intracellular lipid homeostasis is highly dependent on the dynamic balance between lipid biosynthesis and degradation (28). In the liver, acetyl-CoA is catalyzed by ACC to produce malonyl-CoA and FAS catalyzes the synthesis of fatty acids (29). Srebp-1c modulates the expression levels of lipogenic genes such as *ACC* and *FAS*, thereby regulating the synthesis of fatty acids and TG (30). Our findings revealed that RLS inhibit the expression of lipid synthesis-related genes (*ACC, FAS*, and *Srebp-1c*) to reduce hepatic lipid accumulation. CPT-1 and CPT-2 are mitochondrial membrane-associated enzymes that regulate the flow of fatty acids into the mitochondria where β-oxidation occurs (31). PGC-1α participates in the regulation of sugar and lipid metabolism by affecting mitochondrial function. Loss of PGC-1α expression leads to the development of insulin resistance (32). The results from our experiment revealed that RLS treatment significantly increased mRNA levels of lipolysis-related factors CPT-1, CPT-2, and PGC-1α in the livers of rats. These results suggested that RLS promote oxidative decomposition of fatty acids in adipocytes. In summary, RLS maintain the lipid metabolism balance in rats by regulating the expression of key factors involved in fat metabolism.

Lipid metabolic disorders can induce adipocytes to continuously produce pro-inflammatory cytokines and trigger inflammation, further aggravating these disorders (33). Inflammatory cytokines lead to inflammatory reactions and act as pro-inflammatory (IL-1β, IL-6, and TNF-α) and anti-inflammatory (IL-10) cytokines, depending on their roles (34). Biosurfactants modulate the humoral and cellular immune systems. RLS stimulate immune cells to produce pro-inflammatory cytokines (35, 36). Moreover, Andrä et al. (35) stated that RLS from *Burkholderia plantarii* induced human mononuclear cells to produce TNF-α. In this study, we detected a reduction in levels of pro-inflammatory cytokines (IL-1β, IL-6, and TNF-α) in rats following intragastric RLS administration. These results differed from those of previous studies described above. This discrepancy was likely because of the differences in the structure of the RLS used, experimental subjects and intervention times. RLS may inhibit inflammation by reducing concentrations of pro-inflammatory factors; however, the mechanism remains to be elucidated.

Intestinal flora plays a key role in regulating lipid metabolism, immunity, and inflammation (37). Therefore, we analyzed the structure and composition of microflora in rat colon from each treatment group. The results indicated that RLS increased the diversity of intestinal flora in rats. In our previous study, dietary RLS supplementation increased the relative abundance of gut microbiota and promoted the proliferation of beneficial bacteria in broilers, which is similar to the results of this study (15). Additionally, RLS significantly increased the abundance of *Lactobacillus, Roseburia, Ruminococcus-1*, and *Parabacteroides* in the colon. *Lactobacillus* species are known to be probiotics. Specific probiotic *Lactobacillus* strains affect host innate and adaptive immune responses, such as the pro-inflammatory and anti-inflammatory responses of antigen-presenting cells, T cell differentiation, and secretion of antibodies (38–40). Butyrate-producing *Roseburia* species are potential health markers (41). *Roseburia* is a beneficial probiotic in alleviating inflammation in autoimmune diseases (42). *Roseburia* also protects colon epithelial cells from inflammatory damage (43). *Ruminococcus-1* modulates butyrate production by fermenting complex non-digestible polysaccharides, which is thought to be correlated with gut anti-inflammatory responses (44). *Parabacteroides* species have immunoregulatory functions (45). Zeng et al. (46) showed that *Parabacteroides* inhibit systemic inflammatory responses by modulating IL-10 levels and Treg cells. Moreover, *Parabacteroides* exert anti-inflammatory effects by producing short-chain fatty acids (47). Wang et al. (48) demonstrated that *Parabacteroides* attenuate obesity and metabolic dysfunction by producing succinate and secondary bile acids. Altogether, RLS treatment increased the diversity of intestinal flora and improved the relative abundance of beneficial bacteria in rats. However, the effect of rhamnolipids on the gut microbiota is still in the preliminary stage, and its specific mechanism needs to be further studied.

## Conclusion

In conclusion, RLS reduced relative fat weight and adipocyte size, improved the serum lipid profiles, and maintained the lipid metabolism balance by regulating lipid synthesis and degradation-related gene expression, thereby inhibiting inflammation. Additionally, findings from our study demonstrate that RLS increased the diversity of colonic microflora and the relative abundance of beneficial bacteria in rats, which is of great significance for gut health. The specific mechanism needs further study.

## Data Availability Statement

The data presented in the study are deposited in the National Center for Biotechnology (NCBI) repository, https://www.ncbi.nlm.nih.gov/bioproject/, accession number PRJNA818170.

## Ethics Statement

All experiments were conducted in accordance with the Guidelines for the Care and Use of Laboratory Animals of Zhejiang Agriculture and Forestry University and were approved by the Animal Ethics Committee of Zhejiang Agriculture and Forestry University (SYXKzhe 2019-054).

## Author Contributions

BZ and CY conceptualized the study, developed the protocol, and wrote the manuscript. SQ carried out the experiments. YW, RZ, and YX analyzed the data and performed statistical analyses. All authors contributed to the article and approved the submitted version.

## Funding

This research was supported by the Program for Zhejiang Leading Team of Innovation and Entrepreneurship (No. 2020R01015), Key Research and Development Plan Projects of Zhejiang Province (Nos. 2019C02051 and 2020C02032), and National Key Research and Development Program Intergovernmental Cooperation in International Science and Technology Innovation (No. 2018YFE0112700).

## Conflict of Interest

The authors declare that the research was conducted in the absence of any commercial or financial relationships that could be construed as a potential conflict of interest.

## Publisher's Note

All claims expressed in this article are solely those of the authors and do not necessarily represent those of their affiliated organizations, or those of the publisher, the editors and the reviewers. Any product that may be evaluated in this article, or claim that may be made by its manufacturer, is not guaranteed or endorsed by the publisher.
